# Prenatal Diagnosis and Perinatal Outcomes of Posterior Fossa Anomalies in a Tertiary Referral Center: A Five-Year Experience

**DOI:** 10.3390/medicina62071375

**Published:** 2026-07-17

**Authors:** Verda Alpay, Fırat Ersan, Barış Boza, Melike Makul, İbrahim Polat, Aydeniz Aydın Gümüş

**Affiliations:** 1 Department of Obstetrics and Gynecology, Division of Perinatology, Başakşehir Çam and Sakura City Hospital, 34480 Istanbul, Turkey; phratersan@yahoo.com (F.E.); barisboza@hotmail.com (B.B.); melikemakul@gmail.com (M.M.); dripolat@yahoo.com (İ.P.); 2 Department of Medical Genetics, Başakşehir Çam and Sakura City Hospital, 34480 Istanbul, Turkey; aydenizaydingumus@gmail.com

**Keywords:** posterior fossa anomalies, Dandy–Walker malformation, Blake’s pouch cyst, mega cisterna magna, parental counseling, prenatal diagnosis

## Abstract

*Background and Objectives*: Posterior fossa anomalies (PFAs) represent a heterogeneous group of congenital malformations involving the cerebellum and adjacent structures. Prenatal counseling remains challenging because neurodevelopmental outcomes vary substantially according to the specific anomaly. This study aimed to evaluate ultrasonographic and fetal magnetic resonance imaging (MRI) characteristics, associated anomalies, genetic test results, and perinatal and neurodevelopmental outcomes of prenatally diagnosed PFAs in a tertiary care population. *Materials and Methods*: This retrospective study included 115 fetuses diagnosed with PFAs between 2020 and 2024. PFAs were categorized into “simple” (mega cisterna magna, Blake’s pouch cyst [BPC], and arachnoid cyst) and “complex” (Dandy–Walker malformation [DWM], vermian agenesis/hypoplasia, cerebellar hypoplasia, Joubert syndrome, Walker–Warburg syndrome, and pontocerebellar hypoplasia). Maternal characteristics, associated cerebral and extracerebral anomalies, fetal MRI findings, genetic test results, and pregnancy and postnatal outcomes were analyzed. *Results*: Among 115 cases, 44.3% were isolated, and 55.6% were non-isolated. Ventriculomegaly was detected in 35.6% of cases and was significantly more frequent in the complex group (47.9% vs. 14.2%). Fetal MRI was performed in 35.6% (*n* = 41) of cases and demonstrated a 24.3% discrepancy rate with ultrasound, mainly in differentiating DWM from BPC. Genetic testing was performed in 66.9% of cases, revealing chromosomal abnormalities in 15.6% of the total cohort. Complex anomalies, including cerebellar hypoplasia and vermian agenesis/hypoplasia, were more frequently associated with pathogenic copy-number variants and monogenic disorders. The simple group (*n* = 42) had favorable outcomes, with an 89.2% survival rate and neurodevelopmental delay in 15.2% of survivors. In contrast, the complex group (*n* = 73) had significantly poorer outcomes (*p* < 0.001), with a 38.1% survival rate, 42.5% rate of pregnancy termination, and neurodevelopmental delay in 56.3% of survivors. *Conclusions*: This study highlights the persistent diagnostic challenges associated with PFAs and underscores the importance of multimodal imaging and comprehensive genetic evaluation. The proposed simple–complex classification provides a clinically meaningful framework for prognostication and parental counseling. Future research should prioritize prospective multicenter studies with higher rates of comprehensive genetic testing coupled with standardized long-term neurodevelopmental follow-up to refine the understanding of the natural history and prognostic trajectory of these complex brain malformations.

## 1. Introduction

Posterior fossa anomalies (PFAs) constitute a heterogeneous group of congenital malformations affecting the cerebellum and adjacent structures, representing a significant proportion of central nervous system (CNS) abnormalities identified prenatally [1]. Although their exact incidence is unknown, PFAs are encountered in approximately 1 per 5000 live births, highlighting their clinical significance in perinatal medicine [2]. The protracted embryological development of the posterior fossa renders it susceptible to a wide array of structural derangements, ranging from benign mega cisterna magna (MCM) and Blake’s pouch cyst (BPC) to more severe Dandy–Walker malformation (DWM), vermian hypoplasia (VH) and cerebellar hypoplasia [3].

The prenatal diagnosis of these conditions relies heavily on ultrasonography, which serves as the primary screening and diagnostic modality [4]. However, the accurate characterization of PFAs can be challenging due to the subtle and often overlapping sonographic features, particularly in the second trimester [3]. The differential diagnosis between entities such as DWM, characterized by complete or partial agenesis of the cerebellar vermis and cystic dilatation of the fourth ventricle, and BPC, which typically presents with a morphologically normal but upwardly rotated vermis due to the expansion of the Blake’s pouch remnant, remains a significant diagnostic challenge [5]. While fetal magnetic resonance imaging (MRI) is increasingly used as an adjunct to ultrasound, offering superior anatomical detail, it may still yield both false-positive and false-negative results despite its high accuracy [6].

Prenatal counseling in cases of PFA is extremely challenging, as neurodevelopmental outcomes vary widely depending on the specific anomaly [1]. Moreover, the clinical significance of a PFA diagnosis is profoundly affected by the presence of associated anomalies and the underlying genetic etiology [2,4]. PFAs may occur in isolation or alongside other CNS or extra-CNS malformations, which can markedly alter the prognosis [6,7]. Therefore, precise characterization of the PFA subtype, together with thorough evaluation for accompanying structural and genetic anomalies, is essential to provide adequate prenatal counseling [8].

This study aimed to contribute to the growing body of knowledge by retrospectively analyzing our single-center experience with prenatally diagnosed PFAs over a five-year period. We sought to delineate their ultrasonographic characteristics, identify the types of associated anomalies, the spectrum of underlying genetic etiologies, and assess subsequent perinatal outcomes. By integrating these findings, we aimed to improve prognostic accuracy for different PFA subtypes and strengthen the evidence base for clinical management in this challenging area of perinatal medicine.

## 2. Materials and Methods

**Figure 1:**
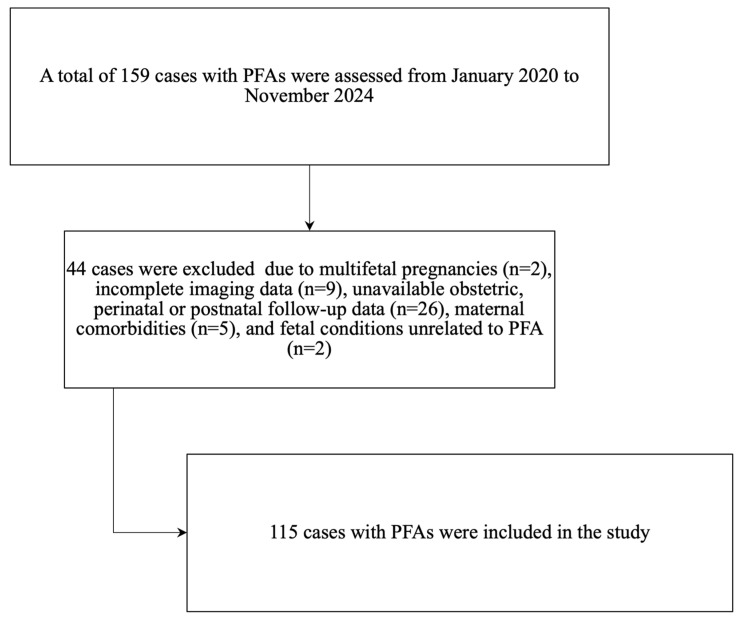
STROBE flow diagram of the study population. Abbreviation: PFAs: posterior fossa anomalies.

We conducted a retrospective cohort study of 115 pregnancies diagnosed with a PFA at our tertiary care perinatology clinic between January 2020 and November 2024. Cases were identified by searching our institution’s electronic medical record and ultrasound database. During the study period, a total of 159 pregnancies with suspected or confirmed fetal posterior fossa anomalies were initially identified. Of these, 2 were excluded because of multifetal pregnancy, 9 because of incomplete imaging data, 26 because of unavailable obstetric, perinatal, or postnatal follow-up data, 5 because of maternal comorbidities (such as preeclampsia, diabetes mellitus, or autoimmune diseases), and 2 because of fetal conditions unrelated to PFA, including confirmed intrauterine infection or fetal anemia. Consequently, 115 pregnancies were included in the final analysis. The patient selection process, including the number of cases excluded for each predefined exclusion criterion, is presented in the STROBE flow diagram in Figure 1.

Prenatal diagnosis was established through targeted neurosonography performed by experienced maternal–fetal medicine specialists. All ultrasound examinations were performed by experienced maternal–fetal medicine specialists using an ARIETTA 850 (Hitachi Medical Corporation, Tokyo, Japan) device (3.5 mHz abdominal transducer). Standard evaluation included a two-dimensional (2D) ultrasound assessment of the fetal brain via transabdominal and, in cases of cephalic presentation, preferably transvaginal approaches. Axial (transcerebellar and transventricular) and sagittal (midsagittal and parasagittal) planes were obtained according to published guidelines [9,10]. The axial transcerebellar view was used to measure cisterna magna (CM), with values >10 mm considered abnormal. The presence of cystic dilatation and communication between the fourth ventricle and the CM was assessed. The cerebellar vermis was visualized in the midsagittal plane, and a craniocaudal length below the 5th percentile was classified as hypoplastic. PFAs were categorized according to the morphological approach proposed by Tortori-Donati et al., and summarized in Table 1 [11].

Fetal MRI was recommended for suspected PFAs to confirm the prenatal neurosonographic diagnosis and identify additional intracranial findings; however, it was performed only when families consented to the examination. MRI examinations were performed between 24 and 32 weeks of gestation. Ultrasound–MRI discrepancy was defined as discordance between prenatal neurosonographic and fetal MRI findings, particularly when the two modalities suggested different diagnostic entities or yielded different preliminary diagnoses of the PFA. Fetal or neonatal autopsy was not performed in our cohort, mainly due to parental preference and cultural reluctance toward postmortem examination. Therefore, final postnatal diagnostic confirmation was based on postnatal clinical evaluation and, when clinically indicated and available, postnatal neuroimaging findings.

For outcome analysis, all diagnosed PFA cases were stratified into two prognostic groups according to previously reported differences in clinical course, associated anomalies, genetic burden, and neurodevelopmental outcomes [8]. The “simple” group included MCM, BPC, and arachnoid cyst, as these entities, particularly when isolated, have generally been associated with more favorable perinatal and neurodevelopmental outcomes. In contrast, the “complex” group included DWM, VH, vermian agenesis, cerebellar hypoplasia, Joubert syndrome, Walker–Warburg syndrome, pontocerebellar hypoplasia, and rhombencephalosynapsis because these anomalies are more frequently associated with additional CNS or extracerebral anomalies, genetic disorders, pregnancy termination, postnatal mortality, and adverse neurodevelopmental outcomes [8]. This classification was performed prior to outcome analysis and was not intended to replace detailed anatomical diagnosis but rather to provide a clinically practical prognostic framework for outcome comparison and parental counseling.

Prenatal genetic testing—including karyotype analysis, chromosomal microarray (CMA) and, when indicated, whole exome sequencing (WES)—was offered to all cases of PFA following genetic counseling. All genetically tested cases underwent conventional karyotype analysis and CMA. Prenatal WES was not routinely performed because of financial constraints and lack of routine coverage; however, postnatal WES was performed in cases requiring further genetic clarification. Amniocentesis or cordocentesis was performed according to appropriate gestational age for patients who opted for invasive diagnostic testing. Postnatal genetic testing was conducted in cases without available prenatal results or when further genetic confirmation was clinically indicated. For CMA, fetal DNA obtained by amniocentesis or chorionic villus sampling, or neonatal DNA obtained from peripheral blood, was analyzed using the Affymetrix CytoScan Optima 315K Array platform version 2023. The data were interpreted using the Chromosome Analysis Suite (ChAS) version 4.5 software. Copy-number variants (CNV) were evaluated according to current publicly available databases, including PubMed, OMIM, DGV, DECIPHER, and ClinGen. Reported genomic coordinates and CNV information were based on the hg38 human reference genome. For postnatal exome sequencing, genomic DNA was isolated from peripheral blood. Library preparation was performed using the Twist Whole Exome Sequencing (WES) Kit (Twist Bioscience HQ, South San Francisco, CA, USA). The coding exons and exon–intron boundary regions of all genes were amplified and analyzed using next-generation sequencing on the MGI DNBSEQ-G400 platform.

Pregnancy outcomes were categorized as termination of pregnancy, live birth, postnatal death, and survival at the last follow-up. Termination of pregnancy was defined as elective termination after prenatal diagnosis of PFA, based on parental decision after multidisciplinary counseling. Live birth was defined as delivery of an infant showing signs of life at birth. Postnatal death was defined as death occurring after live birth during the postnatal follow-up period. Survival at the last follow-up was defined as being alive at the most recent available postnatal assessment. Termination of pregnancy was analyzed and reported as a separate pregnancy outcome and was not combined with postnatal mortality.

A detailed assessment of postnatal medical records and parental interviews was performed to evaluate postnatal follow-up and determine neurodevelopmental status. Neurodevelopmental assessment was performed only among children who were alive at the last available follow-up; infants who died during the postnatal period were not included in the neurodevelopmental outcome analysis. All surviving children were assessed using the Turkish standardized version of the Denver II screening test. Children with abnormal or suspicious Denver II results were referred for formal specialist neurodevelopmental evaluation. Neurodevelopmental status was then classified based on parental reports and medical records. The children included in the neurodevelopmental follow-up ranged in age from 6 months to 4 years, and all remained under clinical follow-up.

Maternal characteristics, gestational age at diagnosis, associated cerebral and extracerebral anomalies, genetic test results, and obstetric and perinatal outcomes were extracted from maternal and neonatal medical records. This study was approved by the institutional review board (approval date: 19 November 2024, approval number: 274), and the requirement for individual patient consent was waived due to the retrospective nature of the analysis.

All statistical analyses were performed using IBM SPSS Version 24.0 (Chicago, IL, USA). Prior to conducting comparisons, the distributions of continuous variables were assessed for normality by examining histogram plots, evaluating skewness and kurtosis values, and performing the Kolmogorov–Smirnov and Shapiro–Wilk tests. Normally distributed continuous variables were expressed as mean ± standard deviation and compared using Student’s *t*-test. Non-normally distributed continuous variables were expressed as median (minimum–maximum) and compared using the Mann–Whitney U test. Categorical variables were expressed as counts and percentages, and comparisons between groups were performed using the chi-square test. Fisher’s exact test was used instead of the chi-square test when expected cell counts were sparse. For key categorical comparisons between the simple and complex groups, effect sizes were reported as odds ratios with 95% confidence intervals. Standardized residuals were examined to determine which categories contributed to statistically significant differences between groups. A *p*-value of <0.05 was considered statistically significant.

## 3. Results

A total of 115 pregnant women diagnosed with fetal PFA between 2020 and 2024 were included in the study. Patients were further classified into simple and complex groups based on the complexity of the diagnosis. Cases considered less complicated and generally associated with more favorable outcomes (MCM, BPC, and arachnoid cyst) were categorized as the “simple” group, whereas diagnoses known to be more complex and to be associated with poorer outcomes (DWM, Joubert syndrome, VH, vermian agenesis, Walker–Warburg syndrome, Pontocerebellar hypoplasia) were assigned to the “complex” group.

Demographic characteristics of the pregnant women are presented in Table 2. The mean maternal age was 29.5 ± 6.05, with a median gravidity of 2 (range: 1–8) and a median parity of 1 (0–7). The mean gestational age at diagnosis was 27.1 ± 5.35, and consanguinity was identified in 16 patients (13.9%). The mean gestational age at delivery and mean birth weight were 33.1 ± 7.03 weeks and 2090 ± 1173 g, respectively. Delivery occurred via cesarean section in 53.1% (*n* = 61) of cases and vaginally in 46.9% (*n* = 54). Regarding pregnancy outcomes, 36 pregnancies (31.3%) were electively terminated after prenatal diagnosis and counseling. The remaining 79 pregnancies (68.7%) resulted in live birth. Of these live-born infants, 30 (38%) died during the postnatal follow-up period, whereas 49 (62%) were alive at the last available follow-up. Among the 49 children who were alive at the last available follow-up, all underwent neurodevelopmental assessment using the Turkish standardized Denver II screening test. Abnormal or suspicious Denver II results were initially observed in 17 children. All children with abnormal or suspicious Denver II screening results underwent formal specialist neurodevelopmental evaluation. In three of these children, the subsequent specialist evaluation was considered within normal limits; however, continued follow-up was recommended. Based on formal specialist evaluation, hospital records, and parental telephone interviews, neurodevelopmental delay was confirmed in 14 children, who remained under follow-up for developmental delay.

Fetal MRI was performed in 35.6% of cases (*n* = 41). In 31 of these cases, MRI and ultrasound findings were consistent, yielding a concordance rate of 75.6% (31/41). Discrepancies between MRI and ultrasonographic evaluations were identified in 10 cases (24.3%). The most frequent discordant pattern was the initial ultrasonographic diagnosis of BPC that was reclassified as DWM on fetal MRI, observed in four cases. In one case, DWM suspected on ultrasound was reclassified as BPC on fetal MRI. In two cases, BPC diagnosed by ultrasound was revised to VH on fetal MRI, whereas in another two cases, VH suspected on ultrasound was reclassified as BPC. In one additional case, DWM suspected on prenatal ultrasound was reclassified as a large retrocerebellar arachnoid cyst on fetal MRI.

Table 3 summarizes the diagnoses, case numbers, perinatal outcomes, and clinical characteristics of patients with fetal PFA. Of the 115 cases, 51 (44.3%) were isolated, whereas the remaining 64 (55.6%) had associated anomalies involving various systems. Among the 64 cases with associated anomalies, 48 (75%) had multisystem involvement, including gastrointestinal, central nervous, cardiovascular, skeletal, and genitourinary systems. Of these 64 patients, 13 (21%) had additional central nervous system anomalies, with corpus callosum anomalies being the most commonly observed (*n* = 11, 17.2%). Among the non-CNS-associated anomalies, cardiac defects were the most frequent, occurring in 12 patients (18.7%), followed by genitourinary anomalies in 7 patients (11%), skeletal anomalies in 4 patients (6.2%), and gastrointestinal anomalies in 3 patients (4.7%). Ventriculomegaly was identified in 41 cases (35.6%), of which 20 were mild and 21 severe.

Comparisons of the demographic characteristics and perinatal outcomes of the cases classified as simple and complex are presented in Table 4. The simple group consisted of patients with diagnoses generally considered less complicated and associated with more favorable outcomes (MCM, BPC and arachnoid cyst), whereas the complex group included diagnoses known to be more severe and associated with poorer outcomes (DWM, Joubert syndrome, VH, vermian agenesis, Walker–Warburg syndrome and pontocerebellar hypoplasia). No statistically significant differences were observed between the groups regarding obstetric history, including gravidity, parity (*p* = 0.334 and *p* = 0.529, respectively). The mean gestational age at diagnosis was 28.1 ± 5.50 in the simple group and 26.3 ± 5.20 weeks in the complex group, with no statistically significant difference (*p* = 0.092). Consanguinity was significantly more frequent in the simple group compared with the complex group (*p* = 0.037). Ventriculomegaly was also significantly more common in the complex group (47.9% vs. 14.2%, *p* = 0.001), with complex PFAs showing higher odds of ventriculomegaly compared with simple PFAs (OR: 5.23, 95% CI: 1.96–13.9). Similarly, associated structural abnormalities were more frequent in the complex group (69.8% vs. 30.9%, *p* = 0.001; OR: 5.00, 95% CI: 2.12–11.23). Genetic abnormalities were identified in 6 of 33 genetically tested cases in the simple group and 12 of 44 genetically tested cases in the complex group; however, this difference was not statistically significant (18.2% vs. 27.3%, *p* = 0.422; OR: 1.69, 95% CI: 0.55–5.26), which may be partly attributable to the relatively limited number of cases who underwent genetic testing in our cohort. Pregnancy outcomes differed significantly between the groups. Termination of pregnancy was more frequent in the complex group than in the simple group (42.5% vs. 11.9%, *p* = 0.001), corresponding to higher odds of TOP in complex PFAs (OR: 5.46, 95% CI: 1.92–15.4). Among live-born infants, postnatal death was significantly more frequent in the complex group (61.9% vs. 10.8%, *p* < 0.001; OR: 13.4, 95% CI: 3.99–44.9), whereas survival at the last follow-up was higher in the simple group. Neurodevelopmental delay was evaluated only among infants who were alive at the last follow-up. Among these survivors, neurodevelopmental delay was more frequent in the complex group than in the simple group (56.3% vs. 15.2%, *p* = 0.006; OR: 7.69, 95% CI: 1.85–33.3).

Among the 115 fetuses included in the study, prenatal or postnatal genetic testing was performed in 77 cases. Genetic abnormalities were detected in 18 cases, corresponding to an overall diagnostic yield of 23.4% among genetically tested cases and 15.6% in the total cohort. Chromosomal aneuploidies were identified in six cases by conventional karyotype analysis: trisomy 13 (*n* = 2), trisomy 18 (*n* = 2), and triploidy (*n* = 2). One pathogenic copy-number variant, de novo 6q23q22.2 deletion, was detected by CMA. The remaining monogenic disorders were diagnosed by postnatal WES. These included fibrodysplasia ossificans progressiva (FOP) in a case with MCM and corpus callosum abnormalities. Four cases of congenital muscular dystrophy–dystroglycanopathy with eye and brain anomalies were presented with vermian agenesis, cerebellar or pontocerebellar hypoplasia, and coexisting anomalies. Additional diagnoses included two cases of Zellweger syndrome, one associated with DWM and the other with BPC; ichthyosis due to an ABCA12 gene mutation in a fetus with rhombencephalosynapsis; CHARGE syndrome due to a CHD7 gene mutation in a case with vermian hypoplasia and multicystic kidneys; and mitochondrial neurogastrointestinal encephalopathy (MNGIE) in a case with MCM. Prader–Willi syndrome, identified in a case with vermian hypoplasia, was suspected based on postnatal clinical findings and subsequently confirmed by DNA methylation analysis. Among these genetic abnormalities, neurodevelopmental delay was observed in surviving infants diagnosed with congenital muscular dystrophy-dystroglycanopathy with eye and brain anomalies, FOP, CHARGE syndrome, Prader–Willi syndrome, and Zellweger syndrome. Results of prenatal/postnatal genetic analyses, including the timing of diagnosis and the diagnostic test leading to the final diagnosis, are presented in Table 5.

## 4. Discussion

### 4.1. Principal Findings

This study provides a comprehensive analysis of 115 prenatally diagnosed PFA cases from a single tertiary care center, contributing valuable insights to the existing literature regarding their diagnostic challenges, associated anomalies, underlying genetic etiologies, and perinatal outcomes. Our principal findings highlight the ongoing difficulties in achieving precise prenatal characterization of these anomalies, the pivotal prognostic role of coexisting structural and genetic abnormalities, and the markedly divergent outcomes observed across different PFA subtypes. The main clinical implication of our findings is that prenatal counseling for PFAs should not rely solely on the anatomical label assigned at the first ultrasound examination. Rather, prognosis appears to be determined by the combined interpretation of posterior fossa morphology and associated structural or genetic abnormalities. In this context, the simple–complex classification used in the present study may serve as a practical counseling framework because it reflects clinically meaningful differences in survival, pregnancy termination, postnatal mortality, and neurodevelopmental outcomes. However, this classification should be interpreted as a prognostic aid rather than a substitute for detailed anatomical and genetic evaluation.

The distribution of PFA subtypes within our cohort reflects the complex case mix typical of a tertiary referral center. Unlike some population-based studies reporting a predominance of MCM and DWM, our series demonstrated a more balanced distribution, with BPC, DWM, vermian agenesis, and MCM each representing approximately 15% of cases [2]. The subtype observed in our cohort is consistent with the findings of Taşdemir et al., who reported that MCM, cerebellar hypoplasia, vermian hypoplasia, BPC, and DWM constituted the majority of their cases [8].

One of the major challenges in the prenatal management of PFAs is achieving an accurate diagnosis, which is essential for guiding appropriate parental counseling. Fetal MRI is increasingly used as an adjunct when a PFA is suspected on ultrasound [12]. In our cohort, fetal MRI was performed in 35.6% of cases, revealing a diagnostic discrepancy with the initial ultrasonographic findings in 24.3% of them. Although this rate is clinically meaningful, it is notably lower than the 41% discrepancy reported between fetal and postnatal MRI in the study by Limperopoulos et al. [13]. This improvement may reflect advancements in both ultrasound and MRI technology, as well as growing expertise in fetal neuroimaging over the past decade. In our cohort, consistent with the existing literature, the diagnostic discordance mainly occurred in the differential diagnosis between DWM and BPC, as well as in distinguishing vermian pathologies from BPC [13]. This suggests that the greatest diagnostic uncertainty occurs when vermian morphology, tegmento-vermian angle, fourth ventricle communication, and posterior fossa cyst configuration are difficult to define sonographically [14]. Therefore, these findings underscore the complementary role of fetal MRI, not as a replacement for high-quality neurosonography, but as a crucial problem-solving tool in equivocal cases to refine the diagnosis and detect subtle associated CNS anomalies that may alter prognosis [6].

The presence of associated anomalies is a major determinant of outcome in fetuses with PFAs [15]. In our study, 55.6% of cases were non-isolated, consistent with previous studies indicating a rate of 58% [1]. This elevated proportion may reflect referral bias, as our institution serves as a tertiary center that receives more complex cases. A key finding of our study was the high prevalence of associated CNS malformations, particularly corpus callosum anomalies, which represented the most frequently identified CNS malformations and were present in 17.2% of our non-isolated cases, consistent with earlier studies [8]. Ventriculomegaly was another common cranial sonographic finding, detected in 35.6% of our total cohort, with 17.4% classified as mild and 18.2% as severe, in line with recent literature [2,16]. Its prevalence was significantly higher in the complex group (47.3%) compared with the simple group (14.6%), highlighting its association with more severe underlying pathology. The presence of ventriculomegaly, particularly when severe, may result from obstructive mechanisms related to the PFA itself or may reflect an associated CNS anomaly [2]. In either circumstance, ventriculomegaly substantially complicates prognosis and management [2].

Our study further demonstrates the substantial diagnostic yield of advanced genetic testing in the evaluation of PFAs. Genetic testing, performed prenatally and postnatally in cases without prenatal results, was conducted in 66.9% of our cohort, and chromosomal abnormalities were identified in 15.6% of all cases. This overall rate is consistent with the literature, which reports variable detection rates depending on the specific anomaly, with higher yields observed in more complex malformations [17]. In our series, complex anomalies such as cerebellar hypoplasia, vermian agenesis, and hypoplasia were more frequently associated with underlying chromosomal anomalies and pathogenic CNVs. This finding aligns with previous reports indicating that fetuses with PFAs and a normal karyotype are often associated with submicroscopic chromosomal aberrations, underscoring the need for CMA when the karyotype is normal [18]. Our results are also in agreement with recent recommendations advocating a sequential genetic strategy, starting with karyotyping and CMA, and proceeding to WES, particularly in non-isolated or complex cases, which has been shown to have a high diagnostic yield of up to 47.5% in this subgroup [19]. The identification of several monogenic disorders in our cohort, such as Zellweger syndrome, CHARGE syndrome, and congenital muscular dystrophy-dystroglycanopathies with eye and brain anomalies, further highlights the critical role of WES in uncovering the underlying etiology, which carries significant implications for prognosis, parental counseling and recurrence risk assessment [19]. Dystroglycanopathy-related disorders represented an important subgroup among the monogenic diagnoses in our cohort. These conditions comprise a genetically heterogeneous group of autosomal recessive disorders caused by defective glycosylation of α-dystroglycan and are frequently associated with variable muscular, ocular, and central nervous system involvement [20]. Severe type A dystroglycanopathies may present prenatally with posterior fossa malformations, cerebellar and vermian hypoplasia or agenesis, brainstem abnormalities, ventriculomegaly, cobblestone lissencephaly, and ocular anomalies [20]. Therefore, a prenatal imaging diagnosis such as Dandy–Walker spectrum malformation or cerebellar hypoplasia may represent the manifestation of an underlying dystroglycanopathy rather than an isolated PFA. In line with previous reports, including prenatal WES-based diagnosis of FKTN-related muscular dystrophy-dystroglycanopathy type A4 in a fetus initially suspected to have familial Dandy–Walker malformation, our findings support the value of WES in complex PFAs when karyotype and CMA are non-diagnostic [21]. Zellweger spectrum disorders should also be considered among the possible monogenic etiologies when PFAs are accompanied by multiple extracranial anomalies. Although cerebellar and PFAs are not specific prenatal ultrasound findings and may be variable in Zellweger spectrum disorders, previous prenatal exome sequencing studies have shown that PEX-related Zellweger syndrome may be diagnosed in fetuses initially presenting with cerebellar or posterior fossa abnormalities together with other systemic anomalies [22]. In our cohort, both Zellweger cases were associated with multiple prenatal anomalies and severe postnatal neurological findings, including seizures, hypotonia, and poor general condition. The detection of pathogenic abnormalities in a substantial proportion of genetically tested cases indicates that PFAs, particularly complex or non-isolated forms, should be regarded not only as structural malformations but also as potential markers of underlying chromosomal or monogenic disorders.

The classification of PFAs into “simple” (MCM, BPC, and arachnoid cyst) and “complex” (DWM, VH, etc.) groups in our analysis revealed a dramatic divergence in both clinical characteristics and perinatal outcomes, representing one of the central findings of our study. The simple group demonstrated overwhelmingly favorable outcomes, with an 892% survival rate and a 15.2% rate of neurodevelopmental delay among survivors. These results broadly align with the meta-analysis by Parisi et al., which reported abnormal neurodevelopment in 2.0% and 4.9% of isolated BPC and MCM cases, respectively [17]. In contrast, the complex group was associated with markedly poor outcomes, including a 42.5% rate of pregnancy termination, a 61.9% rate of postnatal death, and neurodevelopmental delay in 56.3% of survivors. This unfavorable prognosis is consistent with previous studies, which have reported abnormal neurodevelopment in 45.5% and 30.7% of DWM and VH cases, respectively [17]. This clear prognostic dichotomy based on a simple classification scheme can be an extremely valuable tool in parental counseling, providing a more objective framework for discussing expected outcomes and aiding families in informed decision-making. Our findings reinforce existing evidence that isolated simple PFAs such as BPC and MCM are generally associated with favorable outcomes, whereas complex PFAs carry a substantially worse prognosis, particularly when accompanied by additional anomalies [23]. Nevertheless, the simple-complex grouping used in this study and the favorable prognosis of simple PFAs should be interpreted cautiously, particularly when additional anomalies or genetic abnormalities are present. Conversely, complex PFAs appear to represent a heterogeneous but high-risk group in which adverse outcomes may be driven by the combined effect of abnormal posterior fossa development, associated CNS involvement, extracerebral anomalies, and underlying genetic disease.

### 4.2. Clinical Implications

The findings of this study have several clinical implications for the prenatal evaluation of PFAs. First, they support the importance of detailed neurosonography as the primary diagnostic tool, with fetal MRI used as a complementary modality in diagnostically challenging cases, particularly when differentiation between BPC, DWM, vermian abnormalities, and other cystic lesions is uncertain. Second, the higher frequency of associated anomalies, genetic abnormalities, postnatal death, and neurodevelopmental delay in complex PFAs suggests that these cases require a more comprehensive multidisciplinary approach. This should include detailed anatomical survey, fetal MRI when indicated, genetic counseling, and stepwise genetic testing, including CMA and WES when karyotype and CMA are non-diagnostic.

### 4.3. Impact on Prenatal Counseling

From a counseling perspective, our results emphasize that prognosis in PFAs should not be based solely on the initial anatomical label assigned during prenatal ultrasound. Instead, counseling should integrate the specific posterior fossa diagnosis, the presence of ventriculomegaly, associated CNS and extracerebral anomalies, genetic findings, fetal MRI results, and expected postnatal course. The simple–complex classification used in this study may provide a practical framework for communicating broad prognostic differences to families. However, it should be regarded as an exploratory counseling aid rather than an independently validated prognostic model.

### 4.4. Limitations

This study has several limitations. Its retrospective nature introduces the potential for selection and information bias. As a single-center study conducted at a tertiary referral hospital, our cohort may not be representative of the general population and may include a higher proportion of severe and complex cases. Another important limitation of our study is related to the assessment of neurodevelopmental outcomes. Neurodevelopmental status was evaluated using the Turkish standardized version of the Denver II screening test, together with parental reports and available postnatal medical records. Although Denver II is a useful developmental screening tool, it is not equivalent to a comprehensive standardized neurodevelopmental assessment. Another limitation concerns the variability in the age at neurodevelopmental assessment. Although all surviving children underwent Denver II screening, the age range at evaluation was relatively broad, from 6 months to 4 years. Because the sensitivity of Denver II may vary across different developmental stages, neurodevelopmental outcomes may not have been assessed with uniform accuracy across the cohort. In particular, subtle cognitive, language, behavioral, or motor impairments may have been underestimated, especially in younger children. Moreover, reliance on parental reporting may introduce recall and reporting bias. In addition, the exclusion of cases with incomplete imaging data or unavailable obstetric, perinatal, or postnatal follow-up information may have introduced selection bias, as these excluded cases might have differed from the final study cohort in terms of pregnancy outcomes or postnatal prognosis. Another important limitation of our study is the absence of postmortem pathological confirmation and of systematic, sequential antenatal–postnatal imaging correlation. Moreover, postnatal MRI was not performed routinely in all live-born infants but was obtained selectively according to clinical indication. As a result, we could not systematically determine how often postnatal imaging confirmed, refined, or changed the initial prenatal diagnostic impression. Additional anatomical clarification obtained through autopsy or standardized sequential imaging may influence diagnostic accuracy, prognostic interpretation, and the counseling provided after the initial prenatal scan. Finally, the relatively low rate of genetic testing in our population is another important limitation of our study, which may have led to an underestimation of the true burden of chromosomal aneuploidies and monogenic disorders associated with PFAs. Studies with higher rates of comprehensive genetic testing are therefore needed to more accurately delineate the genetic landscape of these anomalies and to clarify their associations with clinical outcomes.

### 4.5. Future Research Directions

Future studies should aim to validate these findings in prospective multicenter cohorts with standardized prenatal imaging protocols, systematic fetal MRI use, and structured postnatal neuroimaging and neurodevelopmental follow-ups. Incorporating autopsy, when acceptable, may also improve radiological–pathological correlation and diagnostic certainty, especially in cases resulting in termination of pregnancy or postnatal death. In addition, broader and more uniform genetic evaluation, including prenatal or postnatal WES, may help clarify the monogenic contribution to complex PFAs and improve genotype–phenotype–radiological correlations. Finally, standardized long-term neurodevelopmental assessment extending into preschool and school age is needed to better define the natural history and true prognostic trajectory of different PFA subtypes.

## 5. Conclusions

Our findings confirm that although prenatal imaging has improved, the precise diagnosis of PFAs remains challenging, warranting a multimodality approach. The presence of associated anomalies and an underlying genetic syndrome are common and represent the most critical determinants of the poor outcomes observed in complex PFAs. Although the proposed simple–complex classification may provide a practical framework for prenatal counseling, prognosis should be individualized according to detailed neurosonographic findings, fetal MRI when indicated, associated CNS and extracerebral anomalies, and genetic results. Given the retrospective design, incomplete genetic testing, and non-uniform neurodevelopmental follow-up, these findings should be interpreted cautiously and validated in prospective multicenter studies with standardized imaging, comprehensive genetic testing, and long-term neurodevelopmental follow-up.

## Figures and Tables

**Table 1:** Ultrasonographic features of the posterior fossa anomalies.

Diagnosis of Malformation	Ultrasonographic Features	Tegmento-Vermian Angle	Torcula	MTS	Keyhole Sign
Mega cisterna magna	• CM > 10 mm • Normal vermis and cerebellum • Closed 4th ventricle	<45 degree	N	-	-
Blake’s Pouch cyst	• Normal size and morphology of vermis • Cystic lesion at the inferior vermis and 4th ventricle • Open 4th ventricle hourglass appearance. • Temporal Regression	Elevated <45 degree	N	-	-
Dandy–Walker malformation	• Enlarged cystic posterior fossa with vermian agenesis/hypoplasia/dysplasia • Cerebellar hypoplasia/agenesis/dysplasia/normal • Inferolateral shift in the coroid plexus of the 4th ventricle	>45 degree	Elevated	-	+
Vermian hypoplasia	• Small vermis for gestational age <5th centile, altered vermian morphology. • Normal torcula and tentorium. Open 4th ventricle (Keyhole appearance)	N	N	-	+
Vermian agenesis	• Vermis agenetic • Cerebellum normal	N	N	-	+
Cerebellar hypoplasia	• Cerebellum <5th centile with normal/enlarged CM	N	N	-	-
Arachnoid cyst	• CM >10 mm • Cerebellum normal • Scalloping of skull/cerebellum vermian complex • Bowing of tentorium may be present	N	N	-	-
Rhombencephalosynapsis	• Cerebellum <5th centile with vermian agenesis and central fusion • Normal/enlarged CM	N	N	-	-
Pontocerebellar hypoplasia	• Cerebellum and brainstem hypoplastic • Brainstem hypoplastic • Vermis normal or hypoplastic	N	N	-	-
Joubert syndrome	• Vermis agenesis or hypoplastic • Cerebellum N or hypoplastic • Brainstem normal	N	N	+	-
Walker–Warburg Syndrome	• Cerebellum and brainstem hypoplastic • Z-shaped brainstem • Vermis hypoplastic or agenetic	N	N	-	-

Abbreviations: MTS: molar tooth sign; N: normal; CM: cisterna magna.

**Table 2:** Demographic characteristics and birth parameters of the patients diagnosed with PFAs.

Parameters		Total (*n* = 115)	Mean (±SD)	Median (Minium–Maximum)
Age (years)			29.5 (±6.05)	
Gravida				2 (1–8)
Parity				1 (0–7)
GA at diagnosis (weeks)			27.1 (±5.35)	
Consanguineous marriage		16 (13.9%)		
Classification of anomaly	Simple	42 (36.6%)		
	Complex	73 (63.4%)		
Extra-abnormality	Isolated	51 (44.3%)		
	Non-isolated	64 (55.6%)		
Type of extra-abnormality	CNS	8 (12.6%)		
	Heart	12 (19%)		
	Cranio-facial	9 (14.3%)		
	Multisystemic	21 (33.3%)		
	Genitourinary	7 (11.1%)		
	GIS	3 (4.76%)		
	Skeletal	4 (6.34%)		
Pre-postnatal genetic assessment	Performed	77 (66.9%)		
	Not performed	38 (33.1%)		
Chromosomal abnormality		18 (15.6%)		
Underwent fetal MRI		41 (35.6%)		
Pregnancy outcome	TOP	36 (31.3%)		
	Live birth	79 (68.7%)		
Postnatal outcome among live births	Postnatal death	30 (38%)		
	Surviving at the last follow-up	49 (62%)		
Neurodevelopmental delay among survivors		14 (28.6%)		
GA at delivery (weeks)			33.1 (±7.03)	
Birth weight			2090 (±1173)	
Gender	Female	59 (51.3%)		
	Male	56 (48.7%)		
Ventriculomegaly	None	74 (64.3%)		
	Mild	20 (17.4%)		
	Severe	21 (18.2%)		
Birth method	CS	61 (53.1%)		
	SVD	54 (46.9%)		

Abbreviations: CNS, Central Nervous System; n, number of patients; SVD, spontaneous vaginal delivery; MRI, magnetic resonance imaging; TOP, termination of pregnancy; GIS: gastrointestinal system. Data are presented as percentages, counts, means (SD), medians, minimums, and maximums.

**Table 3:** Number and clinical characteristics of patients presenting with fetal PFAs based on their diagnosis.

Diagnosis	Number	Ventriculomegaly		Isolated	Other CNS	Non-CNS Anomaly	Genetic Abnormality	TOP	Live Birth	Postnatal Death	Surviving at the Last Follow-Up	ND Delay Among Survivors
		Mild	Severe									
Megacisterna magna	17	2	-	13	2	2	2	1	16	2	14	2
Blake’s pouch cyst	18	4	0	12	0	5	2	2	16	2	14	2
Cerebellar hypoplasia	16	3	9	4	3	9	4	11	5	4	1	1
Dandy–Walker Malformation	17	3	3	3	2	12	1	10	7	6	1	-
Vermian hypoplasia	12	3	1	5	2	5	2	5	7	3	4	2
Vermian agenesis	17	2	2	5	3	9	2	4	13	7	6	5
Joubert syndrome	5	2	2	4	0	1	1	1	4	3	1	1
Walker–Warburg Syndrome	2	0	1	0	1	1	2	1	1	1	0	0
Rhombencephalo-synapsis	2	1	1	0	0	2	1	0	2	2	0	0
Pontocerebellar hypoplasia	2	0	2	0	0	2	1	1	1	0	1	1
Arachnoid cyst	7	0	0	5	0	0	0	0	7	0	7	0
Total (n)	115	20	21	51	13	48	18	36	79	30	49	14

Abbreviations: TOP: termination of pregnancy; ND: neurodevelopmental delay; CNS *:* central nervous system.

**Table 4:** Demographic, clinical, and perinatal characteristics of patients with fetal posterior fossa anomalies, classified as simple or complex.

Parameters		Simple (*n* = 42)		Complex (*n* = 73)	Total (*n* = 115)	*p*-Values ^a^	Effect Size ^f^ (OR; 95% CI)
Age (years) ^b^		29 (±5.94)		29.8 (±6.13)	29.5 (±6.05)	0.469	
Gravida ^c^		2 (1–7)		2 (1–8)	2 (1–8)	0.334	
Parity ^c^		1 (0–5)		1 (0–7)	1 (0–7)	0.529	
GA at diagnosis (weeks) ^b^		28.1 (±5.50)		26.3 (±5.20)	27.1 (±5.35)	0.092	
Consanguineous marriage ^d^		2 (4.76%)		14 (19.1%)	16 (13.9%)	0.037	
Ventriculomegaly ^d^		6 (14.2%)		35 (47.9%)	41 (35.6%)	0.001	5.23 (1.96–13.9)
Extra abnormality ^d^		13 (30.9%)		51 (69.8%)	64 (55.6%)	0.001	5 (2.12–11.23)
Chromosomal abnormality (n/N tested) ^d^		6/33 (18.2%)		12/44 (27.3%)	18/77 (23.4%)	0.422	1.69 (0.55–5.26)
Pregnancy outcome ^d^	TOP	5 (11.9%)		31 (42.5%)	36 (31.3%)	0.001	5.46 (1.92–15.4)
	Live birth	37 (88.1%)		42 (57.5%)	79 (68.7%)		
Postnatal outcome among live births ^d^	Postnatal death	4 (10.8%)		26 (61.9%)	30 (38%)	0.000	13.4 (3.99–44.9)
	Surviving at the last follow-up	33 (89.2%)		16 (38.1%)	49 (62%)		
ND delay ^d,e^		5 (15.2%)		9 (56.3%)	14 (28.6%)	0.006	7.69 (1.85–33.3)
GA at delivery (weeks) ^b^		36.7 (±4.89)		31.1 (±7.16)	33.1 (±6.96)	0.001	
Birth weight (gr) ^b^		2790 (±886)		1739 (±1140)	2114 (±1168)	0.001	
Birth method ^d^		SVD	22 (53.7%)	32 (43.3%)	54 (46.9%)	0.284	
		CS	19 (46.3%)	42 (56.7%)	61 (53.1%)		

Abbreviations: GA: gestational age; n: number of patients; SVD: spontaneous vaginal delivery; CS: cesarean section; TOP, termination of pregnancy; ND: neurodevelopmental delay; OR: odds ratio; CI: confidence interval. ^a^ Level of significance *p* < 0.05. ^b^ Continuous variables distributed normally are expressed as mean and standard deviation and are compared using Student’s *t*-Test. ^c^ Data that were not normally distributed are expressed as median (minimum–maximum) and were compared using Mann–Whitney U test. ^d^ Categorical variables were presented as percentage (%) and count (n) and were compared using the Chi-Square and Fisher’s Exact Test. ^e^ Neurodevelopmental delay was evaluated only among surviving infants, excluding cases that resulted in termination of pregnancy and infants who died during the postnatal period. ^f^ Effect sizes for categorical variables are presented as odds ratios with 95% confidence intervals. Odds ratios indicate the odds of the outcome in the complex group compared with the simple group.

**Table 5:** Genetic assessment results for posterior fossa anomalies.

Results of Genetic Assessment			Timing of Genetic Assessment	Diagnostic Test	Affected Gene	Type of Prenatal PFA	Associated Anomalies	Outcome	Postnatal Findings	n
Trisomy 13		Case 15	Prenatal	Karyotype	47,−,+13	Joubert syndrome	-	Postnatal death	-	2
		Case 68	Prenatal	Karyotype	47,−,+13	Vermian agenesis	Craniofacial	TOP	-	
Trisomy 18		Case 71	Prenatal	Karyotype	47,−,+18	Cerebellar hypoplasia	Craniofacial, skeletal	TOP	-	2
		Case 85	Prenatal	Karyotype	47,−,+18	Vermian hypoplasia	Skeletal, severe fetal growth restriction	TOP	-	
Triploidy		Case 49	Prenatal	Karyotype	69,−	Blake’s pouch cyst	Cardiac, craniofacial	TOP	-	2
		Case 55	Prenatal	Karyotype	69,−	Blake’s pouch cyst	Cardiac, genitourinary, gastrointestinal, craniofacial, fetal growth restriction	TOP	-	
Congenital muscular dystrophy-dystroglycanopathy with brain and eye anomalies, type A, 10			Postnatal	WES	RXYTL1	Vermian agenesis	-	Surviving	Postnatal MRI: vermian agenesis, enlarged cisterna magna Clinical examination: moderate neurodevelopmental delay at 4 years of age, characterized by delayed walking and speech	1
Congenital muscular dystrophy-dystroglycanopathy with brain and eye anomalies, type A, 3		Case 34	Postnatal	WES	POMGNT-1	Cerebellar hypoplasia	Bilateral severe ventriculomegaly	Surviving	Postnatal MRI: cerebellar hypoplasia, bilateral severe ventriculomegaly Clinical examination: Hydrocephalus, moderate neurodevelopmental delay at 2.5 years of age, characterized by inability to walk or speak	2
		Case 59	Postnatal	WES	POMGNT-1	Pontocerebellar hypoplasia	Bilateral severe ventriculomegaly, corpus callosum agenesis	Surviving	Postnatal MRI: pontocerebellar hypoplasia, corpus callosum agenesis, bilateral severe ventriculomegaly Clinical examination: Hydrocephalus, severe neurodevelopmental impairment at 2.5 years of age, characterized by inability to walk or speak and seizures	
Congenital muscular dystrophy-dystroglycanopathy with brain and eye anomalies, type A, 1 (Walker Warburg syndrome)			Prenatal	WES	POMT-1	Cerebellar hypoplasia	Z-shaped brainstem, bilateral severe ventriculomegaly, bilateral cataract	Postnatal death (4 months)	Postnatal imaging: vermian hypoplasia, cerebellar hypoplasia, bilateral severe ventriculomegaly, Z-shaped brainstem Clinical examination: Absent left crystalline lens, right-sided cataract, and enlargement of the right bulbus oculi, hypotonia, multiple seizures	2
Zellweger syndrome	Case 35		Postnatal	WES	PEX-2	Blake’s pouch cyst	Genitourinary, cardiac, musculoskeletal	Postnatal death (7 months)	Clinical examination: Hypotonia, seizures, hepatomegaly, ventricular septal defect, micrognathia, hypertelorism, elevated long-chain fatty acids in the laboratory	1
	Case 105		Postnatal	WES	PEX-1	Dandy–Walker malformation	Genitourinary, cardiac, musculoskeletal	Postnatal death (4 months)	Clinical examination: Hypotonia, seizures, bilateral talipes equinovarus, hydronephrosis elevated long-chain fatty acids in the laboratory	
Fibrodysplasia ossificans progressiva (FOP)			Postnatal	WES	ACVR1	Megacisterna magna	Corpus callosum agenesis	Surviving	Postnatal MRI: corpus callosum agenesis Clinical examination: At 16 months: single phalanges in all fingers except the index fingers, absent toenails, pes planus, not walking independently yet, mild neurodevelopmental delay, no history of seizures.	1
CHARGE syndrome			Postnatal	WES	CHD7	Vermian hypoplasia	Genitourinary, cardiac	Surviving	Postnatal MRI: vermian hypoplasia (<5th percentile) Clinical examination: At 32 months of age, coloboma, postnatal growth restriction, moderate neurodevelopmental delay, complete hearing loss in one ear and 50% hearing loss in the contralateral ear, no seizures	1
Mitochondrial neurogastrointestinal encephalopathy (MNGIE) syndrome			Postnatal	WES	RRM2B	Megacisterna magna (15 mm)	-	Postnatal death (4 months)	Clinical examination: hypotonia, multiple seizures	1
Prader–Willi syndrome			Postnatal	DNA methylation testing	Maternal uniparental isodisomy 15 (upd(15)mat), consistent with Prader–Willi syndrome	Vermian hypoplasia	Craniofacial	Surviving	Postnatal imaging: vermian hypoplasia (<5th percentile) Clinical examination: At 19 months of age; neurodevelopmental delay, poor head control, hypotonia; hearing and visual impairment; surgically treated type 3 CPAM with atypical epithelial proliferation containing mucinous areas; KRAS-positive	1
Harlequin ichthyosis			Postnatal	WES	ABCA12	Rhombencephalosynapsis	Craniofacial, musculoskeletal	Postnatal death (2.5 months)	Clinical examination: bulging eyes, flexed rigid limbs and hypoplastic fingers, eclabium lip	1
De novo 6q23q22.2 del			Postnatal	CMA	De novo 6q23q22.2 del	Blake’s pouch cyst	Cardiac	Surviving	Postnatal imaging: inferior vermian hypoplasia Clinical examination: at 7 months; hearing loss, surgically repaired tetralogy of Fallot, age-appropriate neurological development, no seizures.	1
Total										18

MNGIE: mitochondrial neuro-gastrointestinal encephalopathy disease; TOP: termination of pregnancy; WES: whole exome sequencing; CMA: chromosomal microarray; CC: corpus callosum; CNS: central nervous system; CSP: cavum septum pellicidum; n: number of cases.

## Data Availability

The data that support the findings of this study are available upon reasonable request from the corresponding author.

## Abbreviations

The following abbreviations are used in this manuscript:

PFAs	Posterior fossa anomalies
MRI	Magnetic resonance imaging
BPC	Blake’s pouch cyst
DWM	Dandy–Walker malformation
VH	Vermian hypoplasia
CNS	Central nervous system
MCM	Mega cisterna magna
TOP	Termination of pregnancy
CMA	Chromosomal microarray
WES	Whole exome sequencing
CNV	Copy number variant

## References

[1] Friszer S., Dhombres F., Blondiaux E., Moutard M.L., Garel C., Jouannic J.M. (2018). Patterns of Detection of Fetal Posterior Fossa Anomalies: Analysis of 81 Cases in the Second Half of Gestation. Fetal Diagn. Ther..

[2] Alsehli H., Alshahrani S.M., Alzahrani S., Ababneh F., Alharbi N.M., Alarfaj N., Baarmah D. (2024). Fetal and neonatal outcomes of posterior fossa anomalies: A retrospective cohort study. Sci. Rep..

[3] Yadav A., Singh C., Dagar S., Shastri A., Prakash R., Thakur S. (2022). A myriad of posterior fossa cysts: A single center experience. J. Clin. Ultrasound.

[4] Wüest A., Surbek D., Wiest R., Weisstanner C., Bonel H., Steinlin M., Raio L., Tutschek B. (2017). Enlarged posterior fossa on prenatal imaging: Differential diagnosis, associated anomalies and postnatal outcome. Acta Obstet. Gynecol. Scand..

[5] Ghali R., Reidy K., Fink A.M., Palma-Dias R. (2014). Perinatal and short-term neonatal outcomes of posterior fossa anomalies. Fetal Diagn. Ther..

[6] D’Antonio F., Khalil A., Garel C., Pilu G., Rizzo G., Lerman-Sagie T., Bhide A., Thilaganathan B., Manzoli L., Papageorghiou A.T. (2016). Systematic review and meta-analysis of isolated posterior fossa malformations on prenatal ultrasound imaging (part 1): Nomenclature, diagnostic accuracy and associated anomalies. Ultrasound Obstet. Gynecol..

[7] Long A., Moran P., Robson S. (2006). Outcome of fetal cerebral posterior fossa anomalies. Prenat. Diagn..

[8] Taşdemir Ü., Eyisoy Ö.G., Karaman A., Demirci O. (2025). Ultrasonographic evaluation of fetal posterior fossa anomalies: Six years experience of a tertiary center. J. Clin. Ultrasound.

[9] Malinger G., Paladini D., Haratz K.K., Monteagudo A., Pilu G.L., Timor-Tritsch I.E. (2020). ISUOG Practice Guidelines (updated): Sonographic examination of the fetal central nervous system. Part 1: Performance of screening examination and indications for targeted neurosonography. Ultrasound Obstet. Gynecol..

[10] Paladini D., Malinger G., Birnbaum R., Monteagudo A., Pilu G., Salomon L.J., Timor-Tritsch I.E. (2021). ISUOG Practice Guidelines (updated): Sonographic examination of the fetal central nervous system. Part 2: Performance of targeted neurosonography. Ultrasound Obstet. Gynecol..

[11] Tortori-Donati P., Rossi A., Biancheri R. (2005). Brain malformations. Pediatric Neuroradiology: Brain.

[12] Nguyen T., O’Keane A., Vande Perre S., Chanclud J., le Pointe H.D., Garel C., Blondiaux E. (2025). Fetal imaging of posterior fossa malformations. Pediatr. Radiol..

[13] Limperopoulos C., Robertson R.L., Khwaja O.S., Robson C.D., Estroff J.A., Barnewolt C., Levine D., Morash D., Nemes L., Zaccagnini L. (2008). How accurately does current fetal imaging identify posterior fossa anomalies?. AJR Am. J. Roentgenol..

[14] Kau T., Marterer R., Kottke R., Birnbacher R., Gellen J., Nagy E., Boltshauser E. (2020). Blake’s Pouch Cysts and Differential Diagnoses in Prenatal and Postnatal MRI: A Pictorial Review. Clin. Neuroradiol..

[15] Gandolfi Colleoni G., Contro E., Carletti A., Ghi T., Campobasso G., Rembouskos G., Volpe G., Pilu G., Volpe P. (2012). Prenatal diagnosis and outcome of fetal posterior fossa fluid collections. Ultrasound Obstet. Gynecol..

[16] Garg N., Kumar M., Rai P., Srivastava S.S., Gupta A., Roy Chaudhary S. (2023). Relative prevalence and outcome of fetal posterior fossa abnormality. J. Paediatr. Child Health.

[17] Parisi N., Rizzo G., Cecchini F., Schiattarella A., Khalil A., Mappa I., Timor-Tritsch I., Pilu G., D’Antonio F. (2025). Outcome of apparently isolated fetal posterior fossa anomalies: Systematic review and meta-analysis. Ultrasound Obstet. Gynecol..

[18] Zou Z., Huang L., Lin S., He Z., Zhu H., Zhang Y., Fang Q., Luo Y. (2018). Prenatal diagnosis of posterior fossa anomalies: Additional value of chromosomal microarray analysis in fetuses with cerebellar hypoplasia. Prenat. Diagn..

[19] Juan Z., Cuixia G., Yuanjie C., Yan L., Ling Y., Tiejuan Z., Li W., Jijing H., Guohui Z., Yousheng Y. (2024). Optimal prenatal genetic diagnostic approach for posterior fossa malformation: Karyotyping, copy number variant testing, or whole-exome sequencing?. Eur. J. Med. Res..

[20] Saredi S., Ardissone A., Ruggieri A., Mottarelli E., Farina L., Rinaldi R., Silvestri E., Gandioli C., D’Arrigo S., Salerno F. (2012). Novel POMGNT1 point mutations and intragenic rearrangements associated with muscle-eye-brain disease. J. Neurol. Sci..

[21] Traversa A., Bernardo S., Paiardini A., Giovannetti A., Marchionni E., Genovesi M.L., Guadagnolo D., Torres B., Paolacci S., Bernardini L. (2020). Prenatal whole exome sequencing detects a new homozygous fukutin (FKTN) mutation in a fetus with an ultrasound suspicion of familial Dandy-Walker malformation. Mol. Genet. Genom. Med..

[22] Corsten-Janssen N., Bouman K., Diphoorn J.C.D., Scheper A.J., Kinds R., El Mecky J., Breet H., Verheij J.B.G.M., Suijkerbuijk R., Duin L.K. (2020). A prospective study on rapid exome sequencing as a diagnostic test for multiple congenital anomalies on fetal ultrasound. Prenat. Diagn..

[23] Şeker E., Aslan B., Aydın E., Koç A. (2023). Long-term outcomes of fetal posterior fossa abnormalities diagnosed with fetal magnetic resonance imaging. J. Turk. Ger. Gynecol. Assoc..

